# Sclera color in humans facilitates gaze perception during daytime and nighttime

**DOI:** 10.1371/journal.pone.0249137

**Published:** 2021-03-29

**Authors:** Jessica L. Yorzinski, Amy Harbourne, William Thompson

**Affiliations:** 1 Department of Ecology and Conservation Biology, Texas A&M University, College Station, Texas, United States of America; 2 Department of English, Texas A&M University, College Station, Texas, United States of America; 3 School of Computing, University of Utah, Salt Lake City, Utah, United States of America; University of Tübingen, GERMANY

## Abstract

Species vary widely in the conspicuousness of their eye morphology and this could influence gaze perception. Eyes with conspicuous morphology can enhance gaze perception while eyes with camouflaged morphology may hinder gaze perception. While evidence suggests that conspicuous eye morphology enhances gaze perception, little is known about how environmental conditions affect this interaction. Thus, we investigated whether environmental light conditions affect gaze perception. Human subjects (*Homo sapiens*) were instructed to find direct-gaze faces within arrays of averted-gaze faces or to find averted-gaze faces within arrays of directed-gaze faces. The faces were displayed under conditions simulating nighttime or daytime conditions. Furthermore, the faces had naturally-colored sclera (white) or modified sclera (same color as the iris). Participants were fastest and most accurate in detecting faces during the daytime and nighttime conditions when the sclera were naturally-colored. Participants were worst at detecting faces with modified sclera during the nighttime conditions. These results suggest that eyes with conspicuous morphology enhance gaze perception during both daytime and nighttime conditions.

## Introduction

Gaze perception allows individuals to determine where others are directing their overt attention [1]. Individuals can evaluate if others are looking directly toward them or looking elsewhere within the environment [2, 3]. Furthermore, they can follow the gaze of others to learn about the location of salient objects. Humans and other species follow the gaze of conspecifics to distant locations (reviewed in [4]). Complex forms of social cognition, such as theory of mind, also likely involve gaze perception [1]. Even though gaze perception is vital to many forms of social cognition, we still have much to learn about the factors that affect it.

One factor that could influence gaze perception is eye morphology. Eyes with conspicuous morphology may have evolved to enhance gaze perception while eyes with camouflaged morphology may have evolved to hinder gaze perception [5]. The amount of exposed sclera, sclera color, and iris color are aspects of eye morphology that could contribute to conspicuousness. Humans have unusually large sclera [5] while other primates often have sclera that are less exposed [6, 7]. Humans also have white sclera but many other primates have pigmented sclera [5, 8 but see 7, 9]. Furthermore, human iris color ranges from black to blue [10] while the majority of other primates have irises with shades of brown or yellow [5, 11]. Previous work in humans has demonstrated that eye morphology influences gaze perception [12–19].

Environmental conditions likely interact with eye morphology in influencing gaze perception. This interaction is important because humans often need to function in both daytime and nighttime environments [20, 21]. In fact, preindustrial human populations frequently engaged in nighttime activities (such as foraging, rituals, and socializing; [22, 23]) and these nighttime activities likely involve gaze perception. When humans view objects in low-light conditions, their vision is limited: the threshold visibility, color appearance, sensitivity over time, and visual acuity change compared to viewing the same objects in conditions with bright light [24–26]. For example, humans’ visual acuity (ability to resolve spatial detail) is substantially worse during nighttime compared to daytime [24, 27]. Given human’s limited vision under low-light conditions, we would expect their nocturnal communication signals, including gaze signals, to exhibit properties that facilitate visibility at night (such as high contrast; [28]). We are unaware of any previous work investigating the interaction between environmental light conditions and gaze perception.

We therefore examined human gaze perception under simulated daylight and nighttime conditions. We recorded the eye movements of human (*Homo sapiens*) participants as they searched for directed-gaze faces within arrays of averted-gaze faces or searched for averted-gaze faces within arrays of directed-gaze faces. We used a similar methodology as Yorzinski and Miller [19]. The faces had naturally-colored sclera (white sclera) or modified sclera (sclera color matched the iris color). The faces with modified sclera are a phenotype that is similar to many other primate species; in many primate species, the sclera has a similar color as the iris color (but see [7–9]) or the sclera is minimally exposed [5]. The faces were displayed under conditions simulating nighttime and daytime conditions. The faces that were large and upright, small and upright, or inverted and upright. The large faces were intended to simulate close-range interactions and the small faces were intended to simulate more distant interactions. The inverted faces altered facial configuration but maintained low-level visual features, including contrast and luminance [29]. Our previous work found that humans were faster and more accurate in detecting faces with naturally-colored sclera compared to faces with sclera that matched the iris color under daytime conditions [19]. This study expands upon these results by testing whether participants are also faster and more accurate in detecting faces with naturally-colored sclera (versus faces with sclera that match the iris color) under nighttime conditions. We expected that participants would be fastest and most accurate in detecting faces with naturally-colored sclera under both daytime and nighttime conditions.

## Materials and methods

### Participants

Thirty women and 30 men (between the ages of 18 and 30 years old) participated in this study at Texas A&M University from September 2018 through May 2019. They were of Caucasian ethnicity, and had normal or corrected-to-normal vision. We recruited the participants using emails and flyers, and they earned $20 for their participation. We told participants that the goal of the study was to examine gaze detection. The Institutional Review Board of Texas A&M University (#2016-0575D) approved this study and all participants provided written consent before participating.

### Equipment

We recorded the eye movements of participants using a Tobii Pro screen-based eye-tracker (X2-60; Tobii Technology, Inc., Danderyd, Sweden; accuracy: 0.4 degrees; data rate: 60 Hz; binocular tracking). The eye-tracker was controlled by a laptop computer (Dell Mobile Precision 7510). The experimental stimuli were displayed on a 25” monitor (Dell UltraSharp UP2516D, 2560 x 1440 pixels; Dell Computer Corporation, Round Rock, TX) using Tobii Studio (version: 3.4, Tobii Technology). The eye-tracker noninvasively recorded the precise location of where the participants were directing their overt gaze. In order to minimize head movements, the participants rested their chins on a chin cup (UHCOTech HeadSpot; positioned approximately 60 cm from the screen). Participants were initially told that we were recording the size of their pupils (we debriefed them at the end of the trial to explain that we were recording their eye movements). The luminance of the monitor (sensor positioned in the middle of the monitor and directed toward the screen) displaying a white background was 190 cd/m^2^ and the illuminance of the testing room (sensor positioned in the middle of the monitor and directed toward the participant) was 30 lux (Spectra Cine PhoRad Meter, SC-820, Burbank, California). Before trials began, the eye-tracker was calibrated by having participants fixate 5 points, which appeared sequentially on the monitor. We employed the Tobii Velocity-Threshold Identification filter to classify fixations and saccades based on the velocity of eye movements using the following parameters: I-VT filter,75 ms gap fill-in; average eye selection; 20 ms velocity calculator window, 30 degrees/s I-VT classifier threshold, 75 ms merge adjacent time, and 0.5 degrees merge adjacent angle. Eye movements below the velocity threshold (30 degrees/s) were classified as fixations and those above were classified as saccades.

### Experimental stimuli

The faces we used as stimuli were obtained from the Oslo Face Database [30]. The faces depicted people that were facing the camera (their eyes were either directed or averted) and had neutral expressions. We manipulated the photographs using Adobe Photoshop (Adobe Systems, San Jose, California) and Matlab (Mathworks, Inc., Natick, MA). We removed corneal reflections by replacing them with adjacent colors.

**Fig 1:**
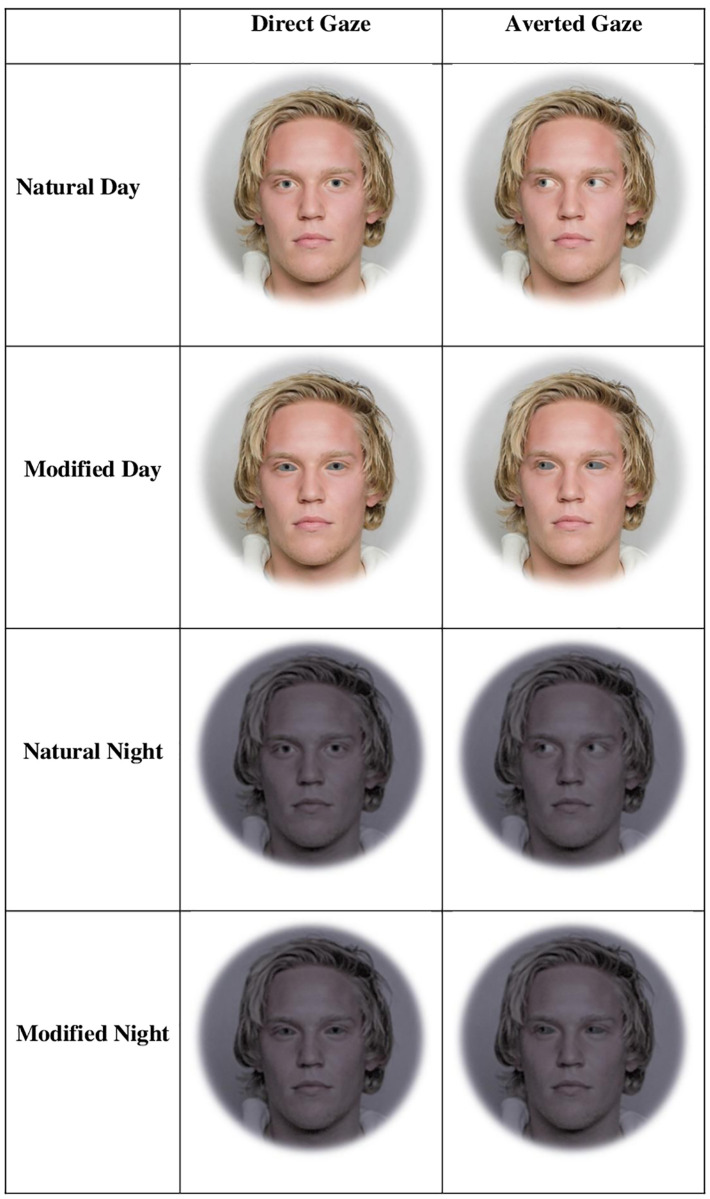
Example stimuli when gaze is direct or averted for a face with light irises for the daytime and nighttime stimuli. The man in the stimuli below gave informed consent for his photograph to be used for scientific purposes [30]. Reprinted from [30] under a CC BY license, with permission from Siri Leknes, original copyright 2014.

**Fig 2:**
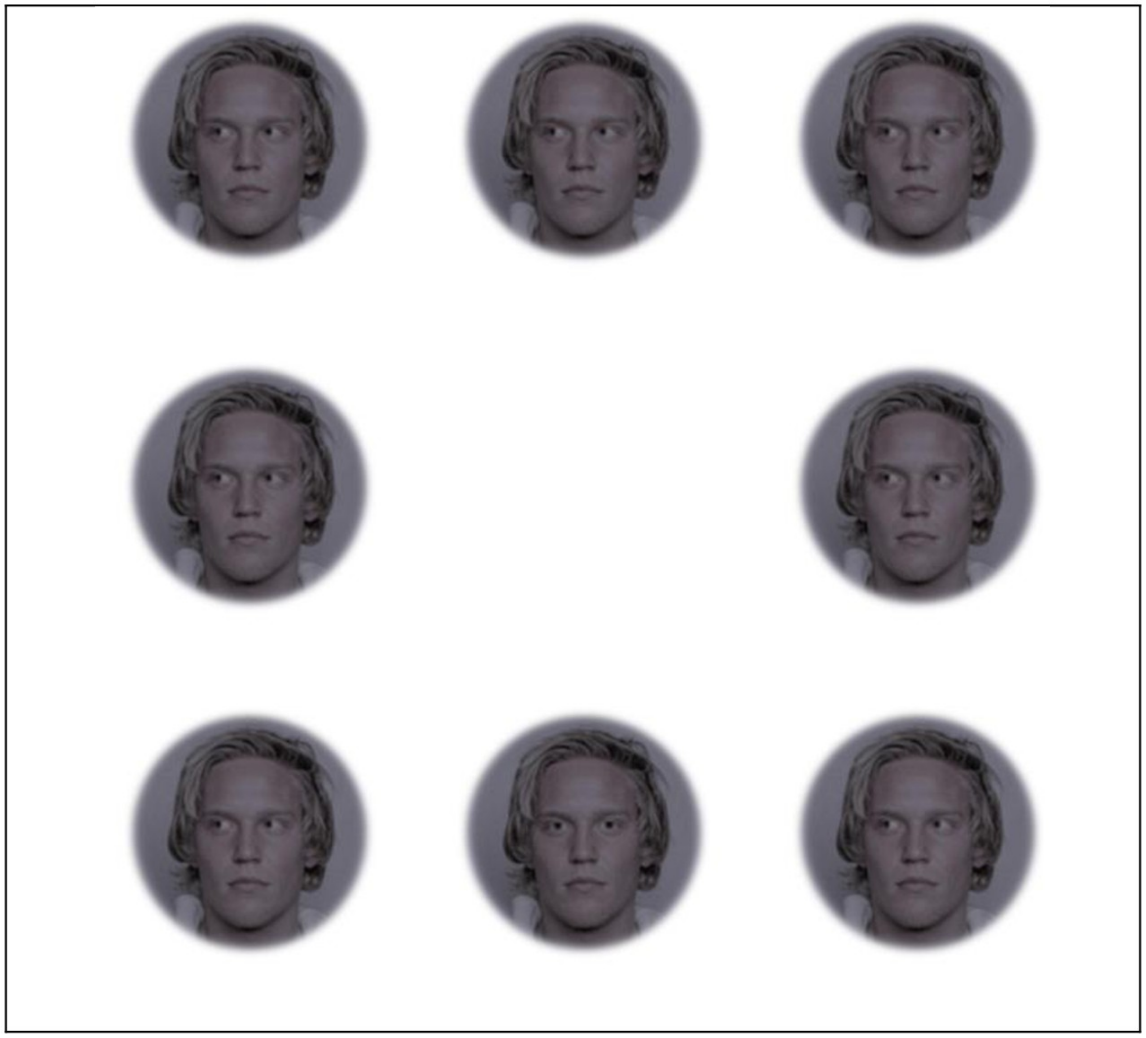
Example of array used in the search task for the Target Directed Natural set at nighttime. The man in the stimuli below gave informed consent for his photograph to be used for scientific purposes [30]. Reprinted from [30] under a CC BY license, with permission from Siri Leknes, original copyright 2014.

We created three blocks of faces each with four sets for two treatments (Figs 1 and 2). The three blocks included faces that were (1) upright and subtended 5.7° (‘large and upright’), (2) faces that were upright and subtended 2.85° (‘small and upright’), and (3) faces that were inverted and subtended 5.7° (‘large and inverted’). Within each of these three blocks, there were four sets of 8-array photographs: (1) face with a direct gaze and natural color within an array of faces with averted gaze and natural color (‘Target Directed Natural’), (2) face with a direct gaze and modified sclera color within an array of faces with averted gaze and modified sclera color (‘Target Directed Modified’), (3) face with an averted gaze and natural color within an array of faces with direct gaze and natural color (‘Target Averted Natural’), and (4) face with an averted gaze and modified sclera color within an array of faces with direct gaze and modified sclera color (‘Target Averted Modified’; Figs 1 and 2). In the faces with modified sclera color, the color of the sclera matched the mean color of the iris. We used the CIELAB color space to make these color modifications because CIELAB is designed to be perceptually uniform for human viewers [31]; it has three axes consisting of L* (lightness), a* (green to red), and b* (blue to yellow). Within each set, 40 different faces were shown. Half of these 40 faces had light-colored irises (subjectively categorized as blue or green; a*: less than two; b*: less than eight; mean ± SE L*: 48.8 ± 0.99) and the other half of these faces had dark-colored irises (subjectively categorized as brown; a*: greater than four; b*: greater than seventeen; mean ± SE L*: 35.5 ± 1.4).

The two treatments included faces that simulated daytime and nighttime conditions. The original images from the face database were photographed under bright lights and were therefore used as the daytime faces. The nighttime faces were created by modifying the daytime faces to simulate nighttime viewing at mesopic luminance levels using the methods outlined in [25]. The use of simulated nighttime viewing was chosen over actual viewing in the dark because it provided us with a good balance of ecological validity and experimental control directly comparable to the daytime viewing cases. Visual sensitivity changes at low light levels due to factors that include differing spectral and magnitude sensitivity of the rods and cones, pupil size, accommodative error, several different neural effects, and others [32]. Perceptually, as luminance levels transition from photopic (only cones active) to mesopic (a mixture of cone and rod activity) to photopic (only rods active), acuity decreases, the ability to differentiate colors decreases, and sensitivity to colors towards the red end of the visible spectrum increases relative to colors towards the blue end of the visible spectrum [24]. Several other subjective effects also occur, including a bluish appearance, an apparent sharpness inconsistent with the decrease in acuity, and noise [25]. For simulated nighttime viewing, we modified luminance and color sensitivity [33].

Because the blue shifting, addition of uncorrelated Gaussian noise, and darkening of display images described in [25] are highly dependent on the experimental conditions, we empirically determined these parameters. Forty images, each with 27 filtered face images (varying in blue shift, darkening, and σ_noise_), were shown to 15 participants (seven women and eight men). For each image, the participants were asked to indicate the top three faces that appeared most similar to how faces appear in moonlight. The parameters were set based on the face that participants selected most often (53% of participants most often selected faces with these parameters as one of their top three choices).

Typical lunar illuminance is around 0.05 to 0.1 lux [34]. Assuming an average skin reflectance of 0.6 for faces and assuming the visible portion of the faces is in direct moonlight, this yields a luminance of the faces of 0.01–0.02 cd/m^2^. We determined the parameterization needed to simulate perception of detail at this light level in ten participants (five men and five women), using the familiar eye chart character recognition task. One of the few studies to empirically measure character legibility under low light levels found a visual acuity of logMAR 0.3 (20/40 Snellen) at 0.4 cd/m^2^ (Lin et al., 2015). We chose to simulate a visual acuity of logMAR 0.4 (20/50 Snellen), since we were simulating a slightly dimmer light level. The parameter controlling visual acuity in the method described in [25] is σ_blur_. Based on informal testing, filtered logMAR chart images were generated for values of σ_blur_ equal to 2.0, 2.5, and 3.0. Each original chart used a different random selection of Sloan characters. For each chart, participants were asked to say the letters of the lowest line that was completely legible to them. They were then asked to read the next lowest line, with the instructions that they should make a reasonable guess so long as they were reasonably certain. The acuity assigned to a particular filtering corresponded to the logMAR value associated with the lowest row in which they could correctly identify a majority of the characters. The σ_blur_ for which the smallest readable line was most often logMAR 0.4 was 2.5. Based on the analyses above, we parameterized a Matlab implementation of the method described in [25] and converted all of the daytime faces to nighttime faces. The Matlab scripts used to create these nighttime images are available at https://github.com/jyorzinski/CreateNightImages.

In the daytime and nighttime faces with natural sclera color, the contrast between the sclera and iris are similarly high (Weber’s contrast mean ± SE: daytime 0.47 ± 0.02; nighttime 0.48 ± 0.02; calculated using the method outlined in [35]); likewise, the contrast between the sclera and pupil are also similarly high (Weber’s contrast mean ± SE: daytime 0.79 ± 0.01; nighttime 0.81 ± 0.02). As expected, in the daytime and nighttime faces with sclera color that matches the iris color, there is minimal contrast between the sclera and iris (Weber’s contrast mean ± SE: daytime 0.10 ± 0.01; nighttime 0.13 ± 0.01); the contrast between the sclera and pupil are also similar but high (Weber’s contrast mean ± SE: daytime 0.64 ± 0.01; nighttime 0.61 ± 0.01). The contrast between the iris and pupil are similar in the daytime and nighttime faces (Weber’s contrast mean ± SE: daytime 0.61 ± 0.01; nighttime 0.65 ± 0.02).

### Experimental procedure

Participants initially performed a practice trial that used photographs of domestic cat faces (*Felis catus*). They were shown photographic arrays that consisted of seven cat faces with directed eyes and one cat face with averted eyes. They were asked to find the cat face with averted eyes as quickly and accurately as possible, and then indicate their response using a custom keypad (model: CP24-USBHID; Genovation, Irvine, CA.). The keypad had eight keys that were arranged in the same configuration as the photographic arrays (a 3 x 3 grid with no key in the middle). The participants also saw photographic arrays in which there were seven cat faces with averted eyes and one cat face with directed eyes (and they had to find the cat with the directed eyes). After indicating each response, a fixation screen briefly appeared (1 s; black dot on a white background) and the next array became visible.

After finishing the practice trial, participants began the experimental task that used photographs of human faces rather than cat faces. During the experimental task, participants were asked to find the face with directed gaze among arrays that consisted of one face with a directed gaze and seven faces with averted gaze (Target Directed Natural & Target Directed Modified). Conversely, participants were also asked to find the face with averted gaze among arrays that consisted of one face with an averted gaze and seven faces with directed gaze (Target Averted Natural & Target Averted Modified). They indicated each response using the custom keypad and a fixation screen briefly appeared (1 s; black dot on a white background) before the next array became visible. Many other studies examining attention use this type of visual search task [36–40]. A given subject completed both treatments (daytime and nighttime; randomized across participants). The blocks and sets were randomized within participants.

### Measurements and statistical analysis

We calculated the amount of time that elapsed between the array appearing and participants fixating on the target (Latency to Fixate Target). We also calculated the amount of time that elapsed between participants fixating on the target and indicating their response (via a keypress; Latency to Press Key After Fixate Target). We performed these calculations by determining whether each fixation coordinate was directed within a rectangular region of interest surrounding the target faces, distractor faces, or neither of them. For each participant, we calculated the mean value of the metrics within each of the four sets (Target Directed Natural, Target Directed Modified, Target Averted Natural, and Target Averted Modified) of the three blocks (large, small, and inverted) for each treatment (daytime and nighttime). We excluded a given array from the analysis if participants never looked at the target or if more than 10% of the gaze data was missing (11% of arrays were excluded using this criteria); this exclusion criteria was set to eliminate cases in which the participants were not performing the task correctly (never looking at the target) or the eye-tracker was malfunctioning (the eye-tracker did not correctly record the participant looking at the target).

We analyzed our data using linear mixed-effects models with repeated measures in SAS (PROC MIXED; Version 9.4; SAS Institute Inc., Cary, NC). The dependent variable was the latency to fixate the target face. The independent variables were the block (large, small, or inverted), set (Target Directed Natural, Target Directed Modified, Target Averted Natural, and Target Averted Modified), treatment (daytime and nighttime), the interaction among block, set and target as well as iris color (light or dark), age, and gender of the participants. Participant identity was included within the models to account for repeated measures. We performed *a priori* contrasts to compare the latency to detect the target face in daytime versus nighttime as well as between the modified sclera and the natural sclera; we performed 24 comparisons and used the false discovery rate correction to evaluate statistical significance [41]. We performed similar repeated-measures mixed linear models using the latency to indicate their response (via a key press) after fixating the target as the dependent variable. In addition, we performed a similar generalized linear mixed model (PROC GLIMMIX; Poisson distribution) using the percentage of correct responses as the dependent variable.

## Results

**Fig 3:**
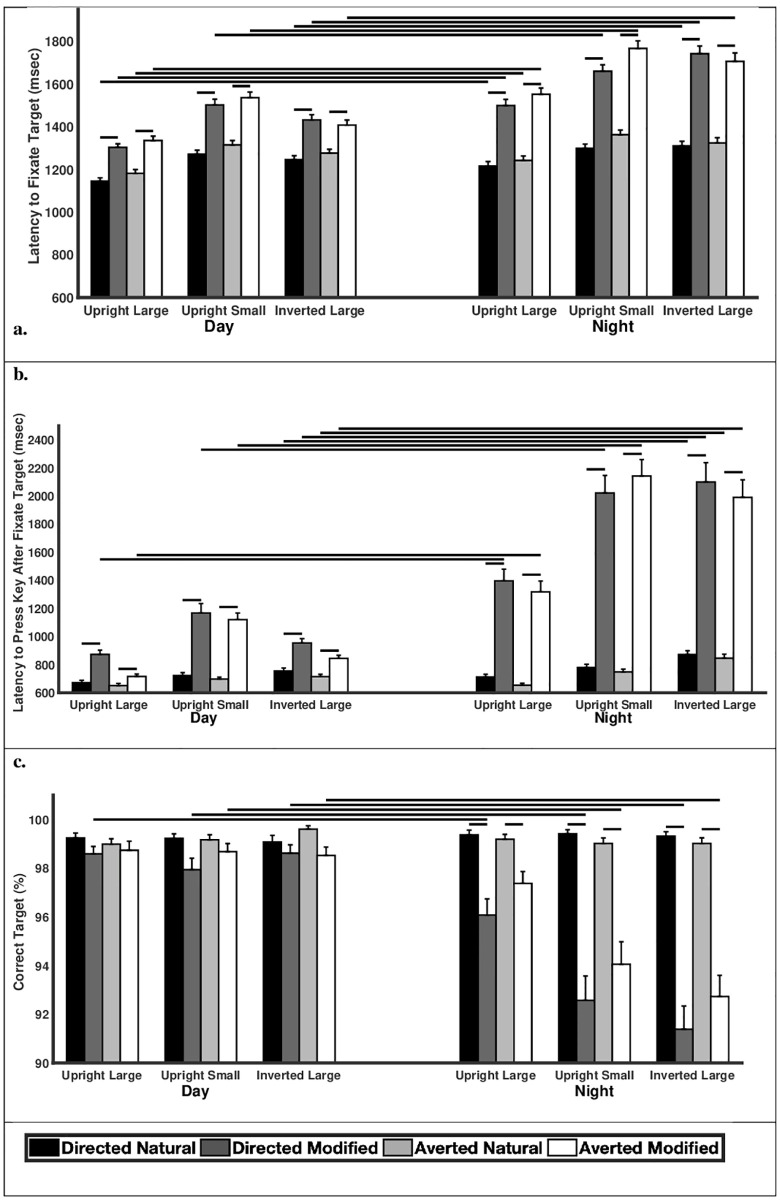
The (a) latency to initially fixate the target, (b) latency to indicate choice (via key press) after initially fixating the target, and (c) percentage of correct responses for large and upright faces, small and upright faces, and large and inverted faces for the daytime and nighttime stimuli. Means and standard errors are shown; horizontal lines indicate planned comparisons that were statistically significant.

Overall, participants were slower to fixate target faces under nighttime versus daytime conditions (F_1,59_ = 202.99, p<0.0001; Table 1: Latency to Fixate Target; Fig 3a). Participants were slower to detect target faces with modified sclera under nighttime compared to daytime conditions. This was the case for faces that were large and upright (directed: F_1,1003_ = 5.80, p<0.0001; averted: F_1,1003_ = 6.40, p<0.0001), small and upright (directed: F_1,1003_ = 4.45, p<0.0001; averted: F_1,1003_ = 5.98, p<0.0001) and large and inverted (directed: F_1,1003_ = 8.35, p<0.0001; averted: F_1,1003_ = 7.94, p<0.0001). When the target faces had naturally-colored sclera, participants were slower to detect the large and upright faces in the nighttime versus the daytime conditions (directed: F_1,1003_ = 2.49, p = 0.013; averted: F_1,1003_ = 2.08, p = 0.038) but they detected small and upright faces at similar latencies irrespective of daytime or nighttime conditions (directed: F_1,1003_ = 0.89, p = 0.37; averted: F_1,1003_ = 1.54, p = 0.12). They were also slower to detect the large and inverted faces in the nighttime versus daytime but only for directed gaze (directed: F_1,1003_ = 2.09, p = 0.037; averted: F_1,1003_ = 1.35, p = 0.18). Participants were faster to detect target faces with light rather than dark iris colors (F_1,59_ = 63.24, p<0.0001).

**Table 1:** The effect of block, set, treatment, iris color, age and gender on the latency to fixate the target and latency to press a key after fixating the target.

	Numerator df, Denominator df	Latency to Fixate Target	Latency to Press Key After Fixate Target
**Overall model**			
Block	2, 118	101.80 (<0.0001)*	109.72 (<0.0001)*
Set	3, 177	275.61 (<0.0001)*	425.98 (<0.0001)*
Treatment	1, 59	202.99 (<0.0001)*	514.66 (<0.0001)*
Block*Set*Treatment	17, 1003	5.46 (<0.0001)*	23.93 (<0.0001)*
Iris Color	1, 59	63.24 (<0.0001)*	386.63 (<0.0001)*
Age	1, 57	43.92 (<0.0001)*	17.20 (0.0001)*
Gender	1, 57	86.31 (<0.0001)*	2.67 (0.11)
**Comparisons**			
Large and Upright			
Target Directed Natural Daytime vs. Directed Natural Nighttime	1, 1003	2.49 (0.013)*	1.13 (0.26)
Target Directed Modified Daytime vs. Directed Modified Nighttime	1, 1003	5.80 (<0.0001)*	7.72 (<0.0001)*
Target Averted Natural Daytime vs. Averted Natural Nighttime	1, 1003	2.08 (0.038)*	0.18 (0.86)
Target Averted Modified Daytime vs. Averted Modified Nighttime	1, 1003	6.40 (<0.0001)*	10.24 (<0.0001)*
Target Directed Natural Daytime vs. Directed Modified Daytime	1, 1003	5.74 (<0.0001)*	5.07 (<0.0001)*
Target Averted Natural Daytime vs. Averted Modified Daytime	1, 1003	5.39 (<0.0001)*	2.05 (0.041)*
Target Directed Natural Nighttime vs. Directed Modified Nighttime	1, 1003	9.05 (<0.0001)*	11.66 (<0.0001)*
Target Averted Natural Nighttime vs. Averted Modified Nighttime	1, 1003	9.71 (<0.0001)*	12.11 (<0.0001)*
Small and Upright			
Target Directed Natural Daytime vs. Directed Natural Nighttime	1, 1003	0.89 (0.37)	1.63 (0.10)
Target Directed Modified Daytime vs. Directed Modified Nighttime	1, 1003	4.45 (<0.0001)*	10.49 (<0.0001)*
Target Averted Natural Daytime vs. Averted Natural Nighttime	1, 1003	1.54 (0.12)	1.17 (0.24)
Target Averted Modified Daytime vs. Averted Modified Nighttime	1, 1003	5.98 (<0.0001)*	12.25 (<0.0001)*
Target Directed Natural Daytime vs. Directed Modified Daytime	1, 1003	7.08 (<0.0001)*	8.40 (<0.0001)*
Target Averted Natural Daytime vs. Averted Modified Daytime	1, 1003	6.76 (<0.0001)*	8.72 (<0.0001)*
Target Directed Natural Nighttime vs. Directed Modified Nighttime	1, 1003	10.63 (<0.0001)*	17.25 (<0.0001)*
Target Averted Natural Nighttime vs. Averted Modified Nighttime	1, 1003	11.2 (<0.0001)*	19.80 (<0.0001)*
Large and Inverted			
Target Directed Natural Daytime vs. Directed Natural Nighttime	1, 1003	2.09 (0.037)*	2.78 (0.0055)*
Target Directed Modified Daytime vs. Directed Modified Nighttime	1, 1003	8.35 (<0.0001)*	13.37 (<0.0001)*
Target Averted Natural Daytime vs. Averted Natural Nighttime	1, 1003	1.35 (0.18)	2.99 (0.0028)*
Target Averted Modified Daytime vs. Averted Modified Nighttime	1, 1003	7.94 (<0.0001)*	14.63 (<0.0001)*
Target Directed Natural Daytime vs. Directed Modified Daytime	1, 1003	6.01 (<0.0001)*	4.66 (<0.0001)*
Target Averted Natural Daytime vs. Averted Modified Daytime	1, 1003	4.24 (<0.0001)*	3.37 (0.0008)*
Target Directed Natural Nighttime vs. Directed Modified Nighttime	1, 1003	12.27 (<0.0001)*	15.25 (<0.0001)*
Target Averted Natural Nighttime vs. Averted Modified Nighttime	1, 1003	10.82 (<0.0001)*	15.01 (<0.0001)*

F values are displayed for the overall model and t values are displayed for the comparisons; p-values are indicated in parentheses and statistically significant comparisons are indicated with an asterisk.

After fixating the correct target face, nighttime and daytime viewing conditions also impacted how quickly participants indicated (via a key press) that they had found the target face (F_1,59_ = 514.66, p<0.0001; Table 1: Latency to Press Key After Fixate Target; Fig 3b). Similar to above, when the target faces had modified sclera, participants were slower to indicate that they found the target under nighttime compared to daytime conditions when the faces were large and upright (directed: F_1,1003_ = 7.72, p<0.0001; averted: F_1,1003_ = 10.24, p<0.0001), small and upright (directed: F_1,1003_ = 10.49, p<0.0001; averted: F_1,1003_ = 12.25, p<0.0001), or large and inverted (directed: F_1,1003_ = 13.37, p<0.0001; averted: F_1,1003_ = 14.63, p<0.0001). Participants were also slower to indicate that they found the target when the faces with naturally-color sclera were large and inverted in the nighttime versus the daytime conditions (directed: F_1,1003_ = 2.78, p = 0.0055; averted: F_1,1003_ = 2.99, p = 0.0028). However, they indicated their responses with similar latencies when the target faces were large and upright (directed: F_1,1003_ = 1.13, p = 0.26; averted: F_1,1003_ = 0.18, p = 0.86;) as well as small and upright (directed: F_1,1003_ = 1.63, p = 0.10; averted: F_1,1003_ = 1.17, p = 0.24). Participants were faster to indicate that they detected the target when the target faces had light rather than dark iris colors (F_1,59_ = 386.63, p<0.0001).

Participants were most accurate in finding the correct target face during daytime compared to nighttime conditions (F_1,59_ = 138.23, p<0.0001; Table 2; Fig 3c). When the target faces had modified sclera, participants were less accurate in identifying the target under nighttime compared to daytime conditions for large and upright faces (directed: F_1,1003_ = 3.74, p<0.0001), small and upright faces (directed: F_1,1003_ = 8.08, p<0.0001; averted: F_1, 1003_ = 6.92, p<0.0001), and large and inverted faces (directed: F_1,1003_ = 10.89, p<0.0001; averted: F_1,1003_ = 8.71, p<0.0001) except when the faces were large and upright with averted gaze (F_1,1003_ = 2.02, p = 0.044). When the target faces had sclera that were naturally colored, participants were equally accurate in identifying the correct target under nighttime and daytime conditions irrespective of whether the faces were large and upright (directed: F_1,1003_ = 0.20, p = 0.84; averted: F_1,1003_ = 0.30, p = 0.76), small and upright (directed: F_1,1003_ = 0.27, p = 0.78; averted: F_1,1003_ = 0.22, p = 0.83) or large and inverted (directed: F_1,1003_ = 0.36, p = 0.72; averted: F_1,1003_ = 0.86, p = 0.39). They were also more accurate in finding target faces with light versus dark irises (F_1,59_ = 155.65, p<0.0001).

**Table 2:** The effect of block, set, treatment, iris color, age and gender on the percentage of correct responses.

	Numerator df, Denominator df	Correct Response
**Overall model**		
Block	2, 118	14.36 (<0.0001)*
Set	3, 177	81.70 (<0.0001)*
Treatment	1, 59	138.23 (<0.0001)*
Block*Set*Treatment	17, 1003	13.19 (<0.0001)*
Iris Color	1, 59	155.65 (<0.0001)*
Age	1, 57	2.96 (0.091)
Gender	1, 57	0.01 (0.91)
**Comparisons**		
Large and Upright		
Target Directed Natural Daytime vs. Directed Natural Nighttime	1, 1003	0.20 (0.84)
Target Directed Modified Daytime vs. Directed Modified Nighttime	1, 1003	3.74 (0.0002)*
Target Averted Natural Daytime vs. Averted Natural Nighttime	1, 1003	0.30 (0.76)
Target Averted Modified Daytime vs. Averted Modified Nighttime	1, 1003	2.02 (0.044)
Target Directed Natural Daytime vs. Directed Modified Daytime	1, 1003	0.96 (0.34)
Target Averted Natural Daytime vs. Averted Modified Daytime	1, 1003	0.37 (0.71)
Target Directed Natural Nighttime vs. Directed Modified Nighttime	1, 1003	4.90 (<0.0001)*
Target Averted Natural Nighttime vs. Averted Modified Nighttime	1, 1003	2.69 (0.0072)*
Small and Upright		
Target Directed Natural Daytime vs. Directed Natural Nighttime	1, 1003	0.27 (0.78)
Target Directed Modified Daytime vs. Directed Modified Nighttime	1, 1003	8.08 (<0.0001)*
Target Averted Natural Daytime vs. Averted Natural Nighttime	1, 1003	0.22 (0.83)
Target Averted Modified Daytime vs. Averted Modified Nighttime	1, 1003	6.92 (<0.0001)*
Target Directed Natural Daytime vs. Directed Modified Daytime	1, 1003	1.9 (0.057)
Target Averted Natural Daytime vs. Averted Modified Daytime	1, 1003	0.71 (0.48)
Target Directed Natural Nighttime vs. Directed Modified Nighttime	1, 1003	10.25 (<0.0001)*
Target Averted Natural Nighttime vs. Averted Modified Nighttime	1, 1003	7.41 (<0.0001)*
Large and Inverted		
Target Directed Natural Daytime vs. Directed Natural Nighttime	1, 1003	0.36 (0.72)
Target Directed Modified Daytime vs. Directed Modified Nighttime	1, 1003	10.89 (<0.0001)*
Target Averted Natural Daytime vs. Averted Natural Nighttime	1, 1003	0.86 (0.39)
Target Averted Modified Daytime vs. Averted Modified Nighttime	1, 1003	8.71 (<0.0001)*
Target Directed Natural Daytime vs. Directed Modified Daytime	1, 1003	0.68 (0.50)
Target Averted Natural Daytime vs. Averted Modified Daytime	1, 1003	1.59 (0.11)
Target Directed Natural Nighttime vs. Directed Modified Nighttime	1, 1003	11.93 (<0.0001)*
Target Averted Natural Nighttime vs. Averted Modified Nighttime	1, 1003	9.44 (<0.0001)*

F values are displayed for the overall model and t values are displayed for the comparisons; p-values are indicated in parentheses and statistically significant comparisons are indicated with an asterisk.

During daytime and nighttime viewing, participants were slower to fixate the correct target face when the face had modified sclera compared to naturally-colored sclera (p<0.0001; Table 1: Latency to Fixate Target; Fig 3a). Participants were also slower to indicate they found the target face when the face had modified sclera rather than naturally-colored sclera (p<0.0001; Table 1: Latency to Press Key After Fixate Target; Fig 3b). Participant accuracy in identifying the correct target was similar (mean accuracy above 97%) in the daytime conditions regardless of gaze direction and sclera color (p>0.05; Table 2; Fig 3c). However, participants were least accurate in identifying the correct target face (large and upright, small and upright, and large and inverted) when the sclera matched the iris color (compared to when the sclera was naturally-colored; p<0.0001; Table 2; Fig 3c).

## Discussion

During daytime and nighttime conditions, participants were faster at fixating target faces with naturally-colored sclera (white sclera) compared to target faces with modified sclera (sclera color that matched the iris color). The target faces simulated both close-up and distant interactions. In addition, after initially finding the correct faces, participants took less time to indicate their responses (via a key press) when the faces had naturally-colored sclera rather than modified sclera, suggesting that they were most certain of their choices when viewing faces with white sclera [42]. We found similar patterns for the inverted faces [43]. These findings suggest that faces with naturally-colored sclera enhance gaze perception in both daytime and nighttime. These results replicate our previous findings that humans are fastest at fixating and indicating their responses when searching for target faces with naturally-colored sclera (versus faces with sclera that match the iris color) in daytime conditions [19].

While faces with modified sclera limit gaze perception during day and night, this effect was particularly pronounced at night. On average, participants took 1.17 times longer to find the faces with modified sclera during the nighttime versus daytime but only 1.04 times longer to find the faces with naturally-colored sclera during the nighttime versus daytime. Similarly, participants spent 1.94 and 1.09 more time to indicate (via a key press) that they found the faces with modified sclera rather than naturally-colored sclera, respectively, during the nighttime rather than daytime. Even though participants were highly accurate in gaze perception (mean accuracy over 90%), they performed the worst when faces had modified sclera during the night. Previous work has also shown that humans are less accurate in judging gaze direction when faces have darkened sclera color [12, 19]. Our results demonstrate that humans are much slower and less accurate in detecting faces with modified sclera during nighttime compared to daytime. Future studies could investigate how subtle changes in sclera color (i.e., sclera colors that are manipulated in small increments) impact gaze detection in both daytime and nighttime.

Faces with naturally-colored sclera may therefore be especially useful in communicating effectively at night. This finding would be expected when considering human night vision [24, 25, 27]. Humans vision is worse under low-light conditions. In fact, a person with 20/10 vision during daylight has 20/300 vision under starlight [24, 27]. Furthermore, visual acuity also depends on contrast such that visual acuity is better when contrast is higher [24]. Given that naturally-colored sclera exhibit higher contrast (white sclera versus black pupil) compared to the modified sclera (blue/brown sclera versus black pupil), we would expect that naturally-colored sclera are easier to resolve. The ability to effectively communicate at night is likely valuable in many contexts. For example, humans are successful hunters at night (Hames, 1979; Hawkes et al., 1991) and conspicuous sclera may be important during these nocturnal hunts for humans to noiselessly communicate with each other [44, 45].

An important limitation of this study is that it simulated nighttime viewing conditions rather than using actual nighttime viewing conditions. While simulated nighttime conditions cannot perfectly mimic actual nighttime conditions, the simulated nighttime condition allowed us to approximate differences in gaze perception between day and night. The simulated nighttime conditions adjusted for luminance and color sensitivities associated with nighttime viewing [24, 25, 33]. Future studies could investigate gaze perception of people when they are outdoors under varying levels of sunlight and moonlight. The combination of highly-controlled laboratory experiments with naturalistic experiments would provide greater insight into the influence of light levels on gaze perception.

Overall, we provided evidence indicating that eyes with naturally-colored sclera enhance gaze perception during both daytime and nighttime conditions. Eye morphology varies widely across species [5–9] and we have a limited understanding of how it impacts gaze perception [44, 46]. Furthermore, inter- and intraspecific differences in visual systems could impact gaze perception relative to eye morphology [47, 48]. Additional work that investigates how environmental conditions, such as lighting or air quality, affect gaze perception across species will provide insight into the factors shaping the evolution of eye morphology.

## Supporting information

S1 Data (XLSX)
